# Stable Speckle Patterns for Nano-scale Strain Mapping up to 700 °C

**DOI:** 10.1007/s11340-017-0317-8

**Published:** 2017-07-28

**Authors:** T.E.J. Edwards, F. Di Gioacchino, H.P. Springbett, R.A. Oliver, W.J. Clegg

**Affiliations:** Department of Materials Science and Metallurgy, University of Cambridge, 27 Charles Babbage Rd, Cambridge, CB3 0FS UK

**Keywords:** Digital image correlation, Gold remodelling, Titanium aluminide, Sub-micron DIC, Nano-DIC

## Abstract

The digital image correlation (DIC) of speckle patterns obtained by vapour-assisted gold remodelling at 200 – 350 °C has already been used to map plastic strains with submicron resolution. However, it has not so far proved possible to use such patterns for testing at high temperatures. Here we demonstrate how a gold speckle pattern can be made that is stable at 700 °C, to study deformation in a commercial TiAl alloy (Ti-45Al-2Nb-2Mn(at%)-0.8 vol% TiB_2_). The pattern is made up of a uniformly sized random array of Au islands as small as 15 nm in diameter, depending on reconstruction parameters, with a sufficiently small spacing to be suitable for nano-scale, nDIC, strain mapping at a subset size of 60 × 60 nm^2^. It can be used at temperatures up to 700 °C for many hours, for high cycle fatigue testing for instance. There is good particle attachment to the substrate. It can withstand ultra-sound cleaning, is thermally stable and has a high atomic number contrast for topography-free backscatter electron imaging.

**Electronic supplementary material:**

The online version of this article (doi:10.1007/s11340-017-0317-8) contains supplementary material, which is available to authorized users.

## Introduction

The spatial resolution of DIC measurements depends primarily on the number density of the speckle pattern that is applied onto the surface of the tested specimen. In recent years, methods have then been developed to progressively reduce the size and spacing of the speckles [1–4]. Creating suitable patterns may be achieved using a variety of methods, including etching [5, 6], electron beam lithography [7], focussed ion beam milling [8], surface abrasion by SiC [9, 10] and sand blasting [11]. It is important that the initial integrity of the sample surface is not lost as preferential weakening of certain microstructural features, in particular grain boundaries, may lead to unrepresentative strain accumulation at such features. Ideally, one requires a microstructure where point features abound, for example, gamma prime precipitates in Ni-based superalloys or stable oxide layers. To ensure the structural integrity of the surface, with applicability across a range of materials, a well-adhered overlay pattern is required.

For high resolution DIC at room temperature, this can be achieved by the deposition of SiO_2_ or Al_2_O_3_ colloids [2, 12, 13], or metal nanoparticles (Pt, Au) [14, 15], electron beam Pt deposition (suitable for small areas only) [16–19] and reconstructed metal thin films [1, 20]. The latter, also referred to as thin film remodelling [1], is where a thin film is heat treated so that it breaks up into an array of particles. For instance, here, a gold thin film is heat treated under steam, causing it to break up to give nanometre-scale gold islands. However, for high temperature testing the methods for obtaining micro- to nano-scale strain mapping are reduced to thermally grown oxide layers [21] and reconstructed metal thin films. It should be noted that there are many examples of DIC strain mapping, but at lower resolutions, at temperatures up to 1400 °C using ceramic powder speckles [22–24].

The resolution of DIC measurements that can be obtained with the above methods is below a few tens of microns. These have then been referred as high-resolution (HR-DIC) methods. This is to distinguish them from the speckle patterns produced by methods such as spray-painting, which are only suitable for coarser scale testing. HR-DIC methods can be further subdivided into two categories: microDIC (μDIC) methods, a term first introduced in [25], with *O*(1 – 100 μm), to which methods employing optical microscopy are generally limited, and submicron resolution methods, referred hereafter as nanoDIC (nDIC), which require imaging resolutions achievable by electron [26] and atomic force [27] microscopies. nDIC is necessary if crystallographic deformation features, such as slip bands, are to be resolved as these are generally spaced a few tens or hundreds of nanometres apart [1]. The choice of HR-DIC method depends on the nature of the substrate and on the testing conditions.

The material investigated here is titanium aluminide, a lightweight replacement for Ni superalloys in gas turbine engines. High cycle fatigue (HCF), up to 10^7^ cycles, is studied at temperatures greater than 700 °C, to understand how cyclic deformation leads to the formation of life-limiting microcracks [28]. At a surface, the TiAl alloy here has a linear microstructure of parallel lamellae traces [29]; this is less convenient for strain mapping [30] than the point feature Ni superalloy γ’ islands. However, there are reports of micro-scale strain mapping of titanium aluminides either without pattern application [31] or in the etched state [32]. The commonly used patterning technique of colloid application is not suitable where testing is being carried out under high temperatures and cyclic loading.

**Fig. 1:**
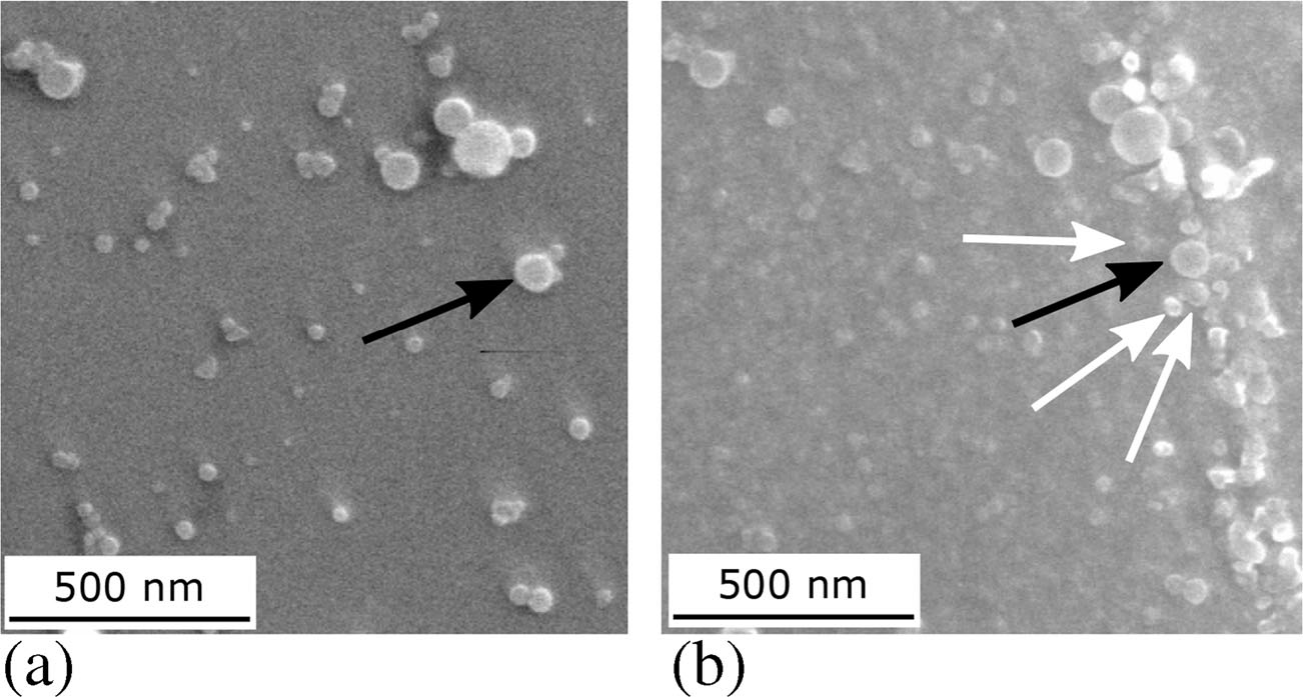
Secondary electron images (through the lens detector) of a SiO_2_ colloid patterned TiAl surface (**a**) before and (**b**) after fatigue cycling at a maximum tensile stress of 425 MPa, *R* = 0.1, 50 Hz and exposure to 700 °C for 1 h. The *black arrows* follow a SiO_2_ colloid whilst the *white arrows* indicate globular oxides grown from thermal exposure, of similar appearance to the colloids

To illustrate this a TiAl specimen with a pattern using colloidal SiO_2_ applied as described elsewhere [13] underwent cyclic loading for 10^6^ cycles at a stress of 425 MPa, approximately equal to the measured threshold stress, and exposure to 700 °C for 1 h, Fig. 1. After testing, colloidal SiO_2_ optimised electron imaging conditions [13] showed a substantial deterioration of the surface pattern to an extent that strain mapping by DIC was no longer possible. This resulted from both the combination of globular oxide growth [33] introducing many more features amongst so that the SiO_2_ particles could no longer be distinguished, together with detachment and loss of some colloid particles.

An alternative approach has therefore been sought using the reconstruction of a thin film into a uniformly random island array. A significant advantage of using Au as the reconstructing element for such patterns is that compared to either oxide layers or colloids of SiO_2_ or Al_2_O_3_, the Au pattern gives rise to significantly better contrast in backscatter electron imaging, which thereby eliminates the effect of deformation-induced substrate topography [1] observed in secondary electron images of a grown oxide, for example. With suitably high contrast imaging conditions, other effects such as crystal orientation channelling contrast are also not observed. Further, the speckle patterns reconstructed from thin films generally display good uniformity resulting from the uniformity of the initially deposited film [1], unlike colloid or nanoparticle deposition that can produce very heterogeneous particle distributions across a sample and relatively low densities of individual, albeit sufficiently small, particles [15].

Here, the remodelling method [1, 4, 34] is developed to give Au reconstructed speckle patterns that are stable at 700 °C. The suitability of the speckle pattern for nDIC is demonstrated for monotonic and HCF testing. A method for making a speckle pattern stable above the temperature limit for Au has also been investigated.

## Experimental Procedures

### Substrate Material

The TiAl alloy, Ti-45Al-2Nb-2Mn(at%)-0.8 vol% TiB_2_, was received in a nearly lamellar condition following casting and HIPping. A more refined lamellar structure was produced by air cooling after 2 h at 1350 °C, and then heating for 8 h at 850 °C, with samples wrapped in Ta foil and encapsulated in quartz glass tubes in 0.15 atm Ar. Samples were electro-discharge machined and ground to 4000 grit, then polished by vibratory polishing (Vibromet 2, Buehler, Germany) in colloidal silica.

### Remodelling

The sample was coated with Au (or Pt, Ag or Pb) using a sputter coater (Emitech K550, Quorum Technologies, UK) and then moved to the apparatus described in [1] for vapour remodelling at 300 °C. Since only a partial reconstruction was obtained, a series of heat treatments were conducted in air, or with the sample wrapped in Ta foil and encapsulated as above to avoid surface oxidation. The description of such heat treatments and their effect on the size, morphology and distribution of the speckles is given in the Results section.

To characterise the stability of the speckle patterns to extended thermal exposure, image correlation of pre- and post-exposure scanning electron microscopy (SEM) images was employed, using commercially available DIC software (DaVis 8, LaVision, Germany). The images were acquired on a dual beam FIB/SEM (Helios NanoLab, FEI, USA). Atomic force microscopy, AFM (Veeco Dimension 3100, Bruker, US), with ultra-sharp tips (TESP-SS, Bruker, US) was used to study the morphology of the speckles.

### Pattern Quality Assessment

There are many measures for pattern quality; such expressions may be classed as local or global. Local indicators evaluate the variability of greyscale values in a subset, a few examples are ones based on intensity gradients from DIC error theory (sum of squares of subset intensity gradient (SSSIG) [35], mean intensity gradient [36], mean intensity of the second derivative [37] and the mean subset fluctuation [38]), and others that consider the entropic variability of subsets [39]. Global indicators provide a single value to instead account for the average pattern quality across a potentially non-uniformly speckled area using particle morphology distribution, or entropic methods (Shannon entropy [40]).

In the current study the suitability of a pattern for image correlation was evaluated using the mean subset fluctuation, (MSF) [38]; a clear benefit of the mean subset fluctuation is its computational ease for an indicator that evaluates the local pixel intensity variations across the whole pattern. It is given by the expression *S*_f_:

1 $$ {S}_{\mathrm{f}}=\frac{\sum_{p\in F}{S}_p}{H\times V}\kern0.5em \mathrm{with}\kern0.5em {S}_p={\sum}_{i=1}^3{\sum}_{j=1}^3\left|{a}_{ij}-\overline{a}\right| $$

where at point *p*, *a*_ij_ is the grey value, $\overline{a}$ the mean of *a*_ij_ for the 3 × 3 pixel subset centred on *p*, and *F* is the image of the speckle pattern considered, of area *H* × *V*. This indicator represents the difference of the pixel intensities in each 3 × 3 subset to the subset average intensity in order to produce a single indicator of image quality describing the average extent of local intensity variations. For comparability between patterns, the MSF is normally calculated from 8-bit greyscale images.

**Fig. 2:**
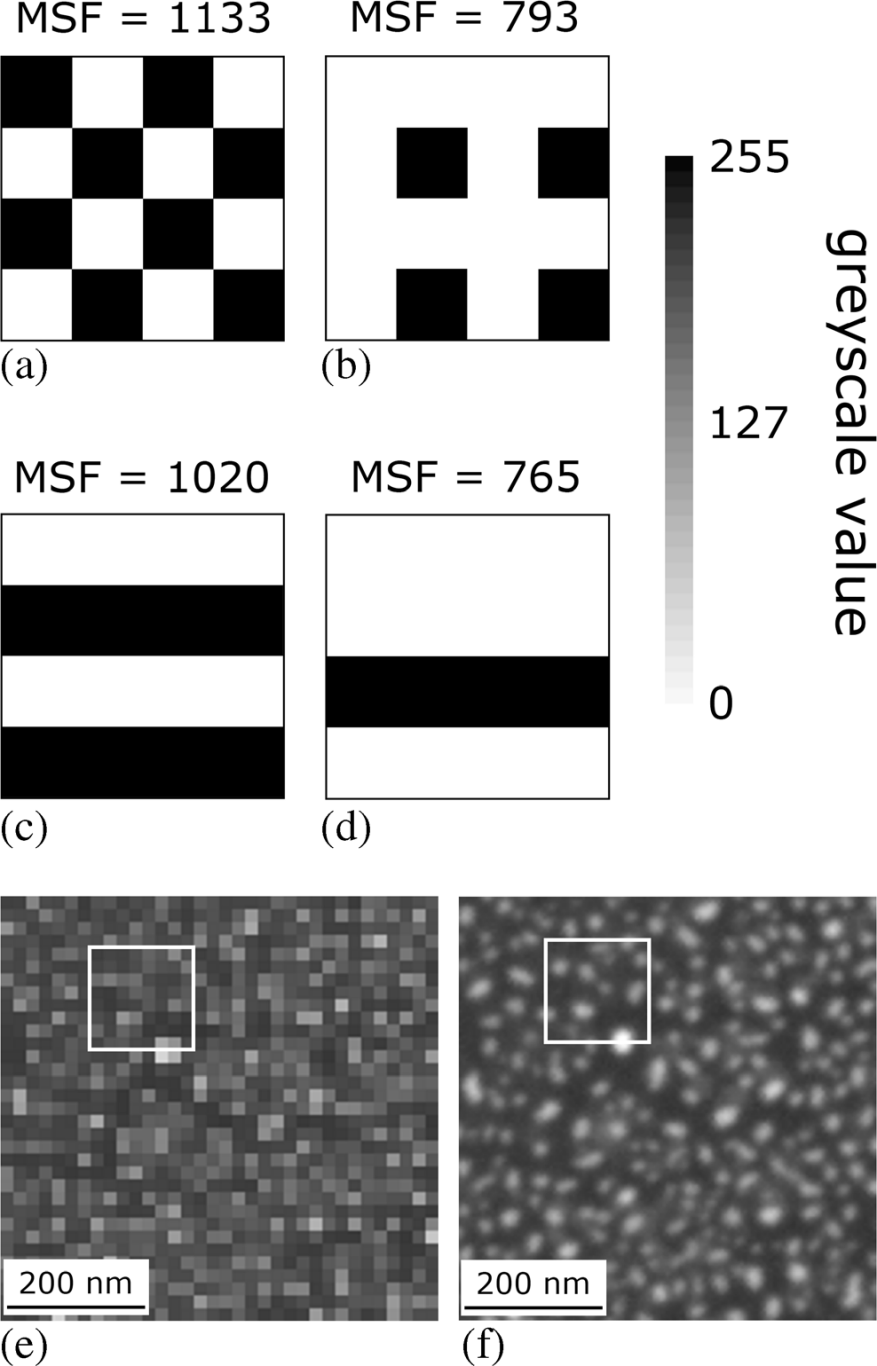
(**a**–**d**) model 4 × 4 pixel array repeat units with the associated large field MSF values, demonstrating that the MSF of patterns may be very high despite being highly repetitive and hence unsuited for DIC strain mapping. (**e**, **f**) backscatter electron images of the same area of an Au speckle patten at a resolution of (**e**) 1024 × 884 pixels and (**f**) 4096 × 3536 pixels. The *white boxes* in (**e**) and (**f**) delineate equivalent spatially sized correlation subsets of 8 × 8 and 32 × 32 pixels, respectively

For higher MSF values there must be large variations, and hence steep gradients, between immediate pixel neighbours. However this is not a sufficient condition for successful strain mapping. Indeed, the regular pixel arrays with binary 8-bit intensities in Fig. 2(a–d) generate very high MSF values, ~10 times higher than those of the experimental patterns in the present or other studies [38], despite lacking the randomness required for a unique strain mapping solution [30]. Further, the combined effect of image resolution and intended subset size is not effectively accounted for by the MSF. Noise levels in strain maps are strongly dependent on the minimum subset size [1]. If two cases are considered, both resulting in the same strain mapping resolution at the same magnification and dwell time, where one employs a 1024 × 884 pixel image and an 8 × 8 minimum subset size, Fig. 2(e), whilst the other is at 4096 × 3536 pixels and a 32 × 32 minimum subset, Fig. 2(f), the latter case is found to present a worse MSF value, by a factor of 2 to 3, despite a nominally increased accuracy of strain mapping and lower noise [30]. This disconsiders effects like increased sample drift for longer image capture times. Additionally, the lower resolution image is likely to suffer from pixel locking [41].

Recently, interest has been in dynamic subset selection [42] where the correlation algorithm is capable of selecting the most appropriate subset size locally, varying this as necessary across the pattern, and hence generating a spatially varying resolution of the calculated displacement field.

With these considerations in mind, a second pattern quality indicator, the subset size dependent mean subset fluctuation (SSDMSF), is proposed here as an adaptation to the MSF equation of Hua et al. [38]. The SSDMSF accounts for a destined DIC subset size in determining its pattern quality value, as follows:

2 $$ \begin{array}{ccc}\hfill {S}_{\mathrm{SSDMSF}}=\frac{\sum_{p\in F}{S}_{p,SSD}}{H\times V}\hfill & \hfill \mathrm{with}\hfill & \hfill {S}_{p,SSD}=\frac{\sum_{i=1}^n{\sum}_{j=1}^n\left|{a}_{ij}-\overline{a}\right|}{A_{\mathrm{subset}}}\hfill \end{array} $$

for a square subset of size *n* × *n* pixel^2^; all other notation is as above. The SSDMSF sums intensity differences to the mean subset intensity across the whole subset size rather than the 3 × 3 nearest neighbour consideration of the normal MSF, followed by normalisation by the number of pixels in the subset (*A*_subset_).

### Monotonic Compression and High Cycle Fatigue Testing

Cuboids for compression testing 4 × 4 × 8 mm^3^ in size were electro-discharge machined and ground on all faces to 4000 grit, with one large face vibratory polished in colloidal silica. Compression testing was carried out *ex situ* on a 25 kN screw machine (Tinius Olsen, U.K.) at a strain rate of 10^−3^ s^−1^, to several strain increments. Samples were heated in air by a halogen lamp hoop heater (Heraeus Noblelight GmbH, Germany) stable to ±0.5 °C of the setpoint, placed around the cuboid, with a type K thermocouple junction for PID control spot welded to the middle of another side face.

Fatigue samples had a 2 × 2 × 8 mm^3^ square cross-section gauge prepared equivalently to the compression cuboids. HCF testing at 50 Hz in tension, *R* = 0.1 and load-controlled, was performed on a 100 kN servo-hydraulic machine (Mayes, UK).

Between all mechanical testing and SEM imaging steps, the testpieces were handled in air, using polyacetal tweezers. A custom built sample holder for the test specimens ensured mechanical clamping of the testpiece for SEM imaging of the speckle pattern; this noticeably reduced temporal drift compared to alternatives such as silver dag fixation or, worse, carbon tape. Imaging was carried out as an up to 10 × 10 grid on the dual beam FIB/SEM in high contrast backscatter mode, to be stitched after correlation [43].

Image correlation of pre- and post-deformation SEM images was performed on DaVis as above to characterize the performance of the applied speckle pattern.

## Results

### Reconstructed Speckle Patterning on a TiAl Alloy

#### Au speckle for high temperature use

**Fig. 3:**
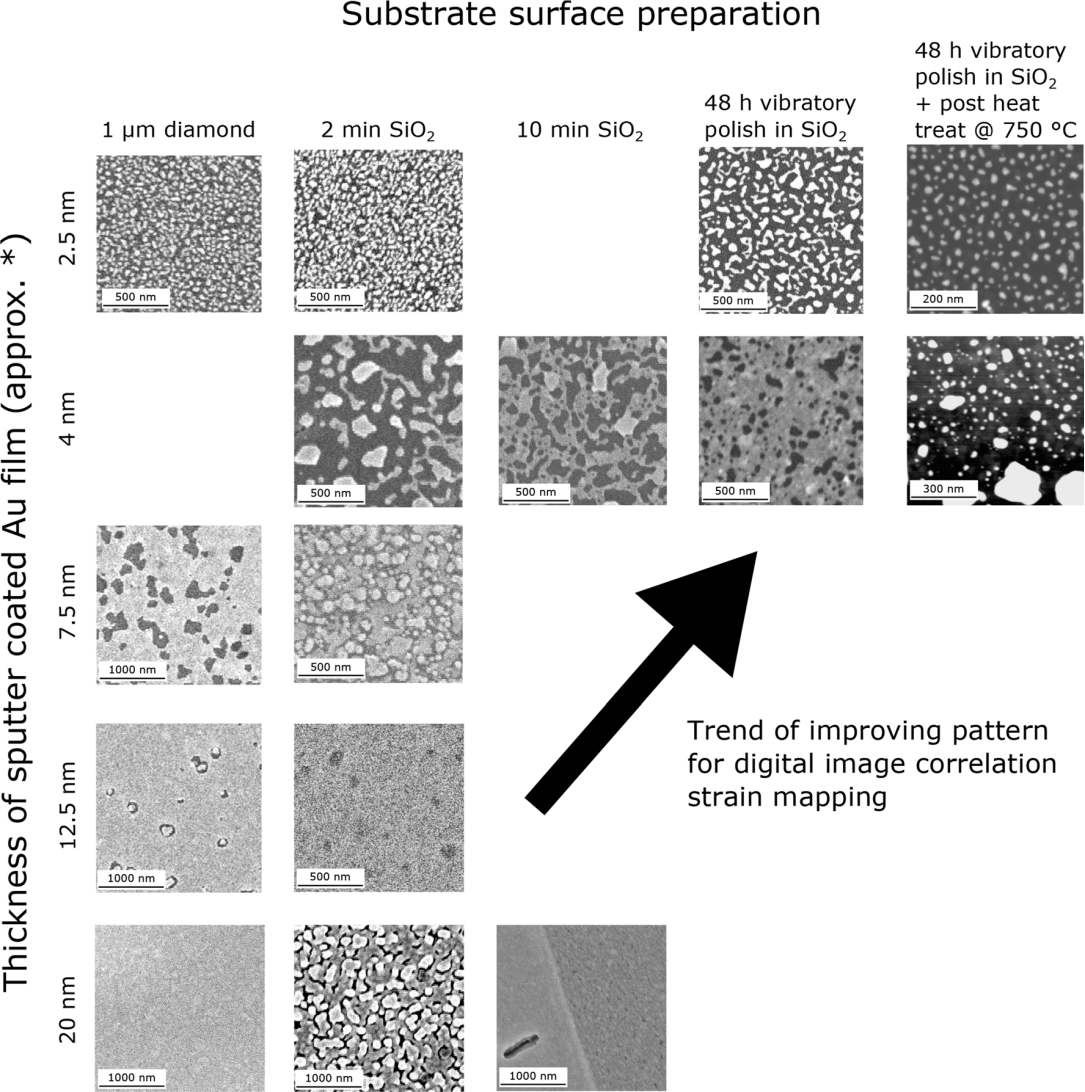
Matrix of backscatter electron images illustrating the results of Au thin film remodelling for 1 h in flowing H_2_O vapour upon varying initial Au film thickness and TiAl substrate surface preparation. ‘post heat treat @ 750 °C’ refers to the heat treatments introduced in the current work that proceed preliminary vapour reconstruction. * the Au thin film thicknesses are estimates based upon the nominal deposition rate determined by the sputter coater manufacturer

A selection of patterns illustrative of the trends resulting from variations in the thickness of the sputtered Au thin film and temperatures of the different reconstruction steps on TiAl is given, Fig. 3; changes to the polishing medium and polishing time affecting the pattern generation are also in the figure. Even after long times, the vapour-assisted remodelling at 300 °C only produced an initial break-up of the Au film giving a crazy-paving appearance. However, an additional treatment at 750 °C in air gave a refined island morphology, which coarsened with longer treatment times. In the absence of a preliminary subdivision of the gold by vapour-assisted remodelling, only a coarse structure could be achieved at 750 °C, unsuitable for nDIC. Subsequent holding at 700 °C stabilised the island morphology against further reconstruction for high-temperature testing.

The TiAl alloy investigated here has a two-phase α_2_-Ti_3_Al/γ-TiAl lamellar structure. Ideally, the speckle pattern should be reconstructed in a similar way on both phases, that is to say preferential aggregation of the Au on top of the α_2_-Ti_3_Al phase should not occur and further the speckle size and spacing should be uniform on both phases. However, reconstruction conditions that enabled a uniform speckle pattern also on the small TiB_2_ boride particles, could not be identified.

**Fig. 4:**
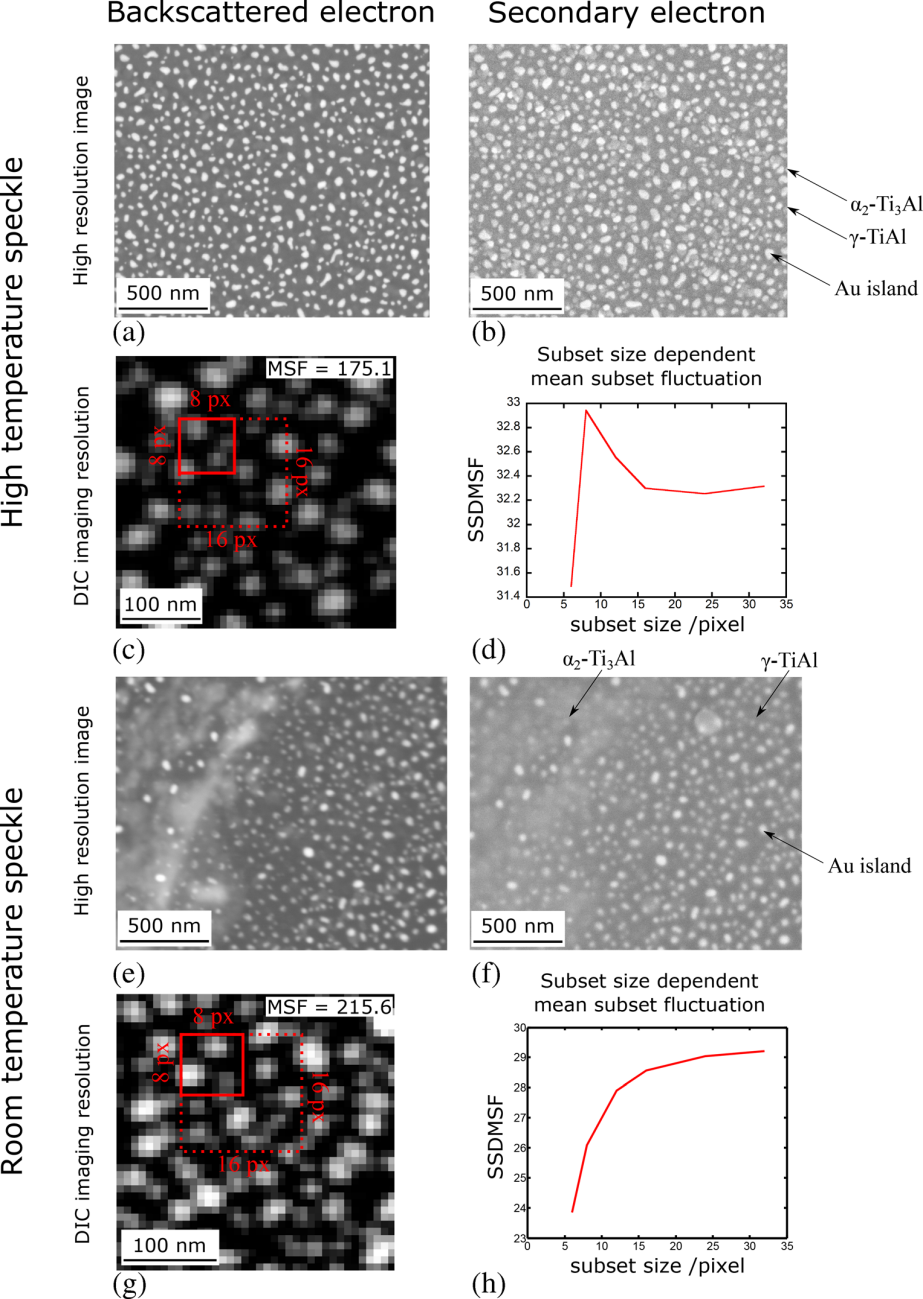
High resolution (**a**, **e**) backscatter and (**b**, **f**) secondary electron images of the reconstructed Au speckle pattern suitable for testing at 700 °C and 25 °C, respectively. Backscatter electron images at DIC image acquisition resolution for both patterns are given in (**c**, **g**) respectively, with example correlation subset sizes overlaid in *red*. Values for the quality of both patterns, measured by the MSF and the SSDMSF, are given in (**c**, **d**) and (**g**, **h**). In (**b**) some oxide intergrowth between the Au islands is visible. The locations of γ-TiAl and α_2_-Ti_3_Al lamellae are indicated, however they are not discernable in (**a**), which is the detector condition employed for DIC strain mapping at 700 °C. Some differential Au reconstruction is observed between the two phases in (e), the imaging condition employed at 25 °C. For the high temperature suitable pattern, the SSDMSF is highest for the 8 × 8 pixel subset

The best patterns were obtained by heating a 2.5 nm thick Au layer for 1 h in flowing H_2_O vapour with the sample at 300 °C, followed by 25 min at 750 °C and then 1.5 h at 700 °C in air. This gave a homogeneous, random array of Au islands 15 – 50 nm in diameter, Fig. 4(a, b) in high resolution and Fig. 4(c) at the resolution for DIC strain mapping, with an average MSF value of 175.1. This value is greater than that of the SiO_2_ colloid patterns considered in Fig. 1 at an identical magnification, or other DIC speckle patterns previously characterised by MSF [38]. The SSDMSF for the high temperature speckle is plotted in Fig. 4(d) against subset size; from this the 8 × 8 subset emerges as the size with the highest average local variability in pixel intensity, with 6 × 6 faring poorly and sizes above 8 × 8 being relatively indistinct. The histogram of pixel intensities for this pattern is given in the supplementary material.

**Fig. 5:**
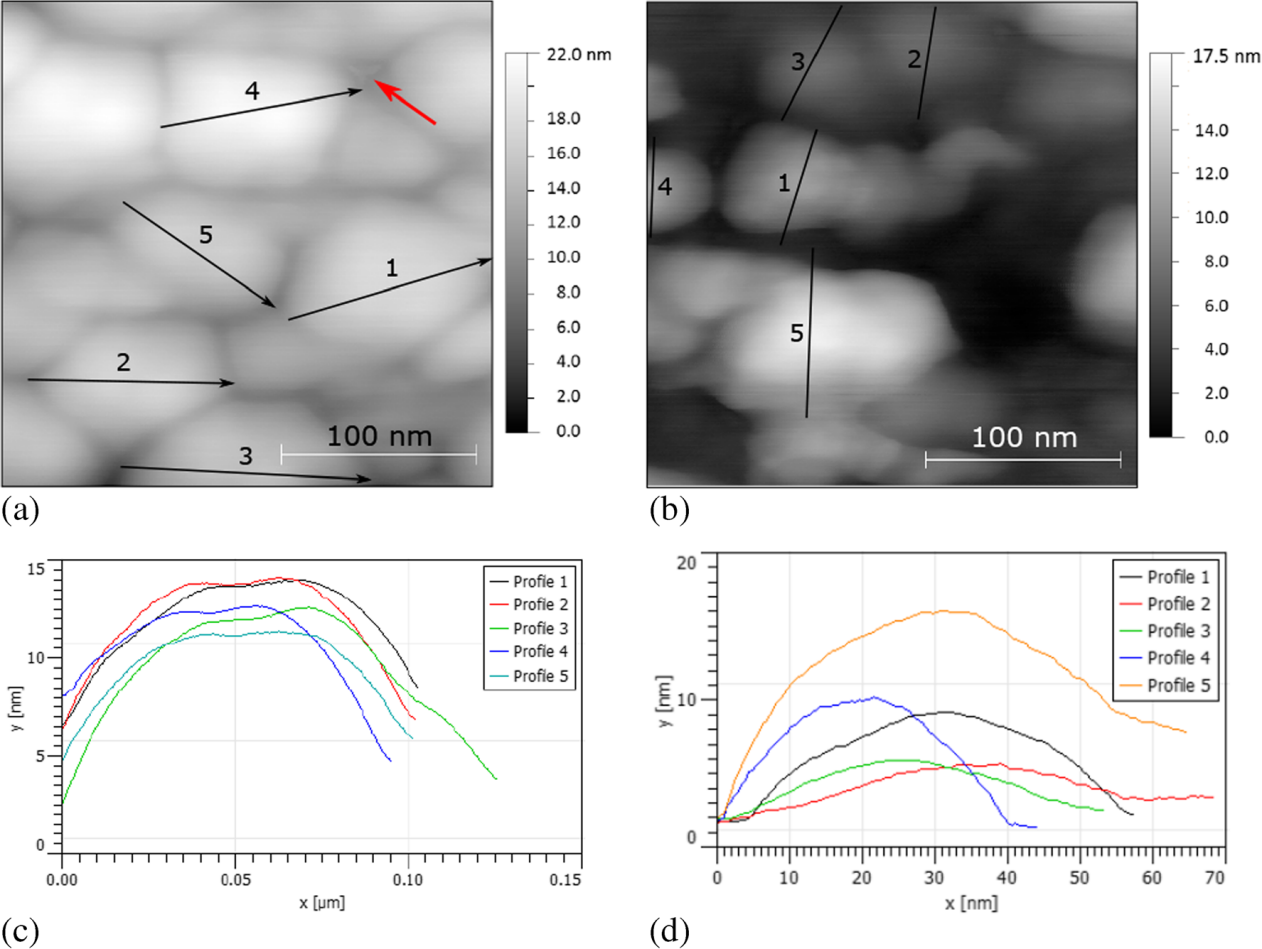
(**a**, **b**) topographical maps by tapping mode AFM of the Au speckle patterns reconstructed in air and in vacuo, respectively, for use at 700 °C and 25 °C, respect. Distinct faceting of the islands in the former is evidenced by the lineplots in (**c**), whilst the Au speckle pattern for use at room temperature displays globular, spatially separated islands (**d**). The *red arrow* in (**a**) indicates a scanning artefact associated with non-linear response effects of the ultrasharp tip necessary to resolve such islands

The morphology of the gold speckles, as measured by AFM, Fig. 5(a, c), showed some evidence of crystallographic faceting; the profiles in Fig. 5(c) in particular show a flat top and inclined sidewalls.

To date, approximately 50 test specimens have been tested using a reconstructed Au pattern. All showed very high and useable speckle uniformity across the entire areas, in excess of 50 mm^2^ per sample including head regions for gripping, provided that prior to Au deposition the surfaces were well cleaned following vibratory polishing and no water, acetone or ethanol liquid residue remained.

#### Au speckle for room temperature use

The previous pattern was found to be unsuitable for room temperature testing due to cracking and spalling of the surface oxide generated upon Au remodelling above 700 °C. To prevent oxide growth the sample was encapsulated in quartz, backfilled with Ar to 0.15 atm, after Au deposition and before reconstruction. Without the possibility for water vapour-assisted reconstruction, and cooperative Au island and TiAl oxide growth, speckle production techniques were limited to furnace heat treatments. Matrices of Au film thickness, furnace temperature and time conditions were similarly produced as above; a successful pattern was obtained after 15 min at 750 °C then 1 h at 700 °C, Fig. 4(e–h). This pattern was somewhat coarser than the previous, with Au particle sizes of 50 nm to 200 nm. AFM also indicated a different morphology of the gold islands, Fig. 5(b, d). No clear faceting was observed, rather the gold islands appeared as curved mounds with a single apex. These were also further apart than those reconstructed in air. This vacuum reconstructed pattern was not suitable for testing in air at 700 °C due to further oxide growth-assisted reconstruction, but functioned well at room temperature. It had an average MSF of 215.6.

### Stability and Imaging of Au Speckle Pattern for nDIC Strain Mapping

**Fig. 6:**
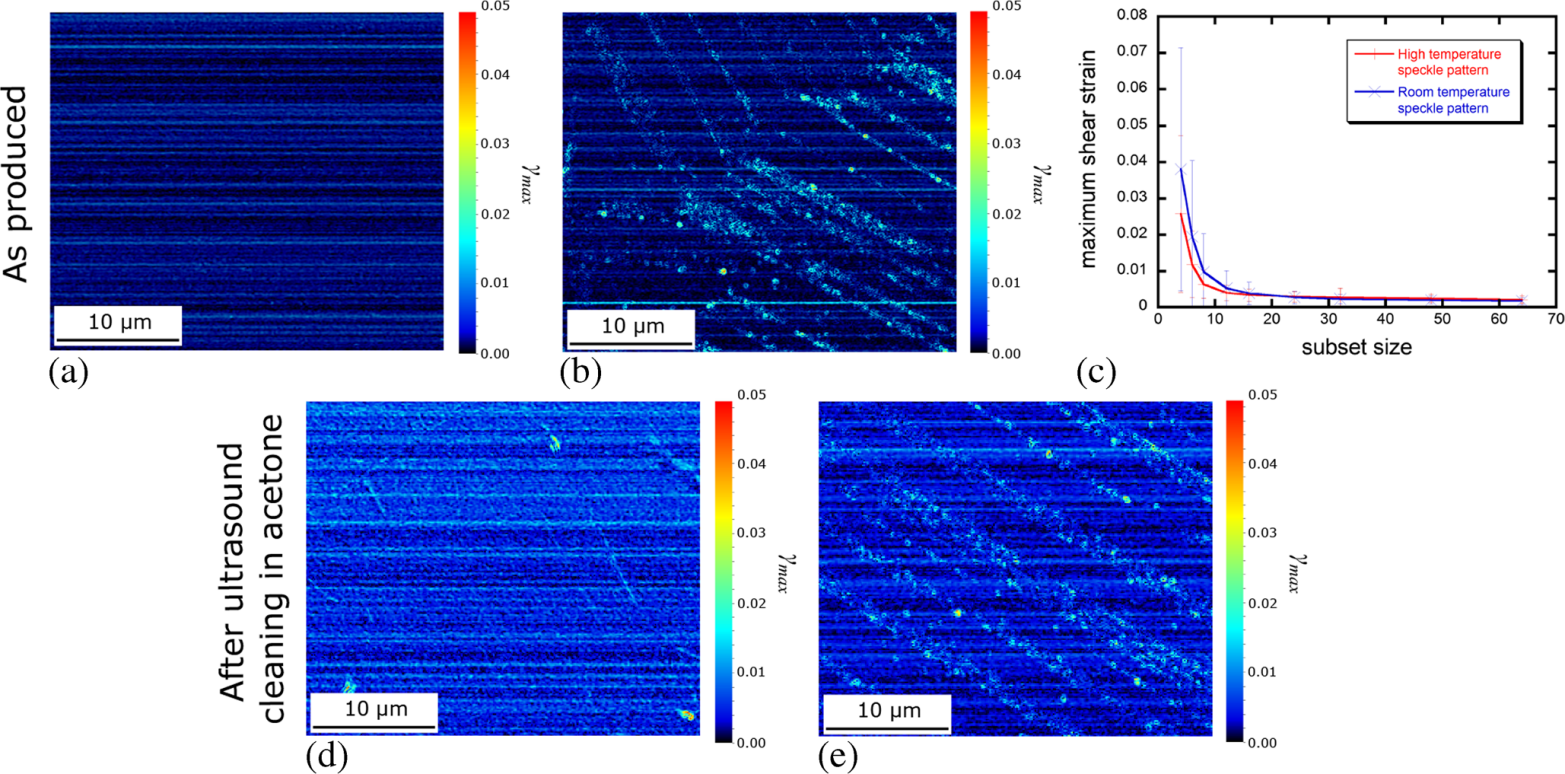
Maps of maximum shear strain of repeatedly imaged regions of the (**a**) high temperature suitable speckle pattern and (**b**) oxide-free pattern, to determine noise levels inherent to the imaging setup and pattern chosen. The average and standard deviation of noise for varying square subset sizes (side length, in pixels) is graphed in (**c**) for both speckle patterns. In (**b**) the effect of differential Au reconstruction between phases is visible as varying noise levels; this is not in (**a**). Equivalent noise maps resulting from ultrasound cleaning in acetone for 20 min are given for (**d**) the high temperature suitable speckle pattern and (**e**) the oxide-free pattern. The average and standard deviation of noise in the maximum shear strain from ultrasound cleaning are as follows: (**d**) 0.6 ± 0.3% and (**e**) 0.4 ± 0.3%; hence no significant increase in noise is observed

**Fig. 7:**
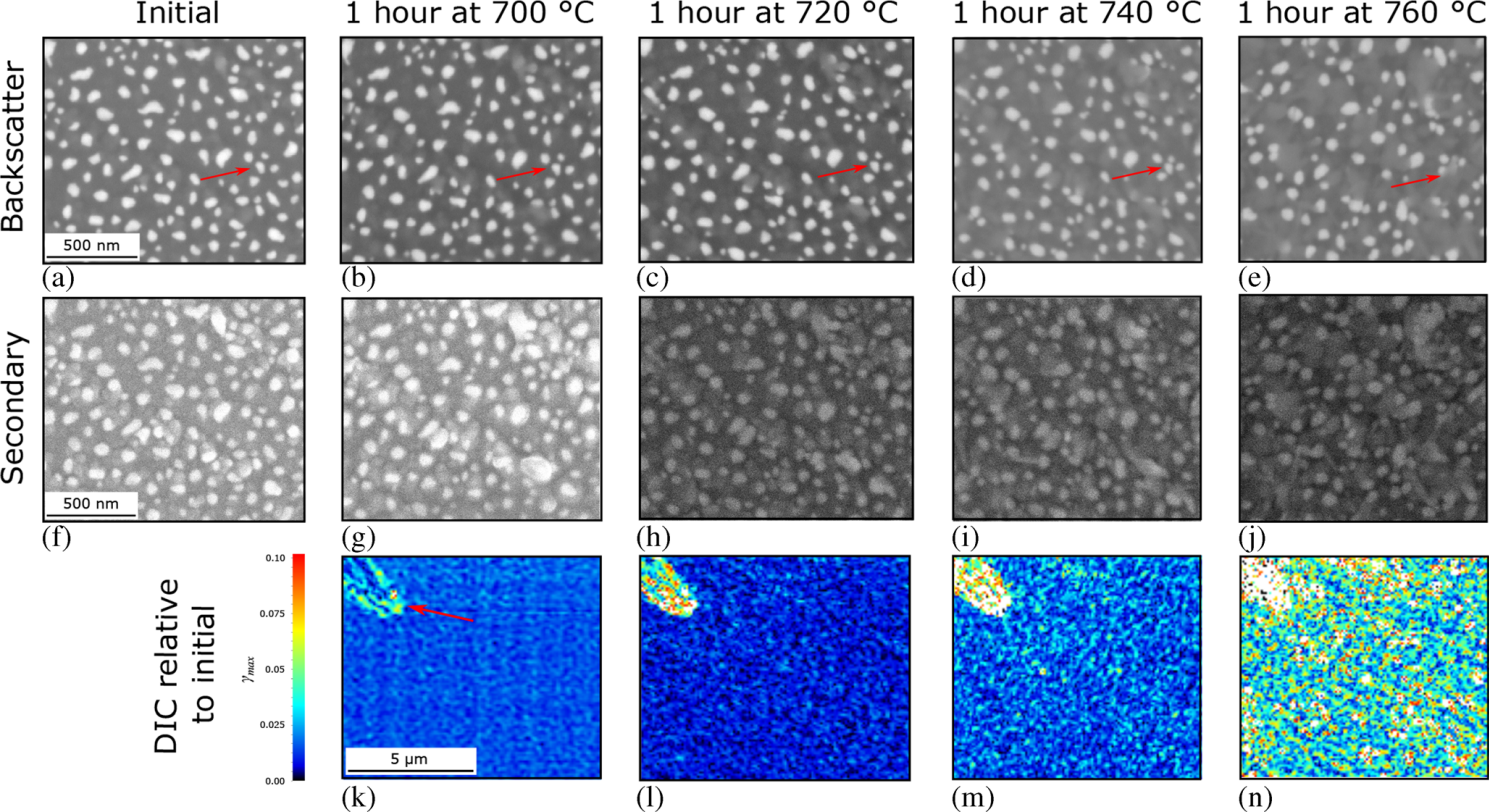
(**a**–**e**) backscatter and (**f**–**j**) secondary electron images of a same region of the high temperature speckle pattern following successively higher temperature holds. Until 740 °C good spatial stability of the Au islands is observed by low maximum shear strain from DIC (**k**–**n**) relative to the initial state, produced from correlation of the backscatter electron images at 4000× magnification, despite the increasing oxide content evidenced by secondary electron imaging

**Fig. 8:**
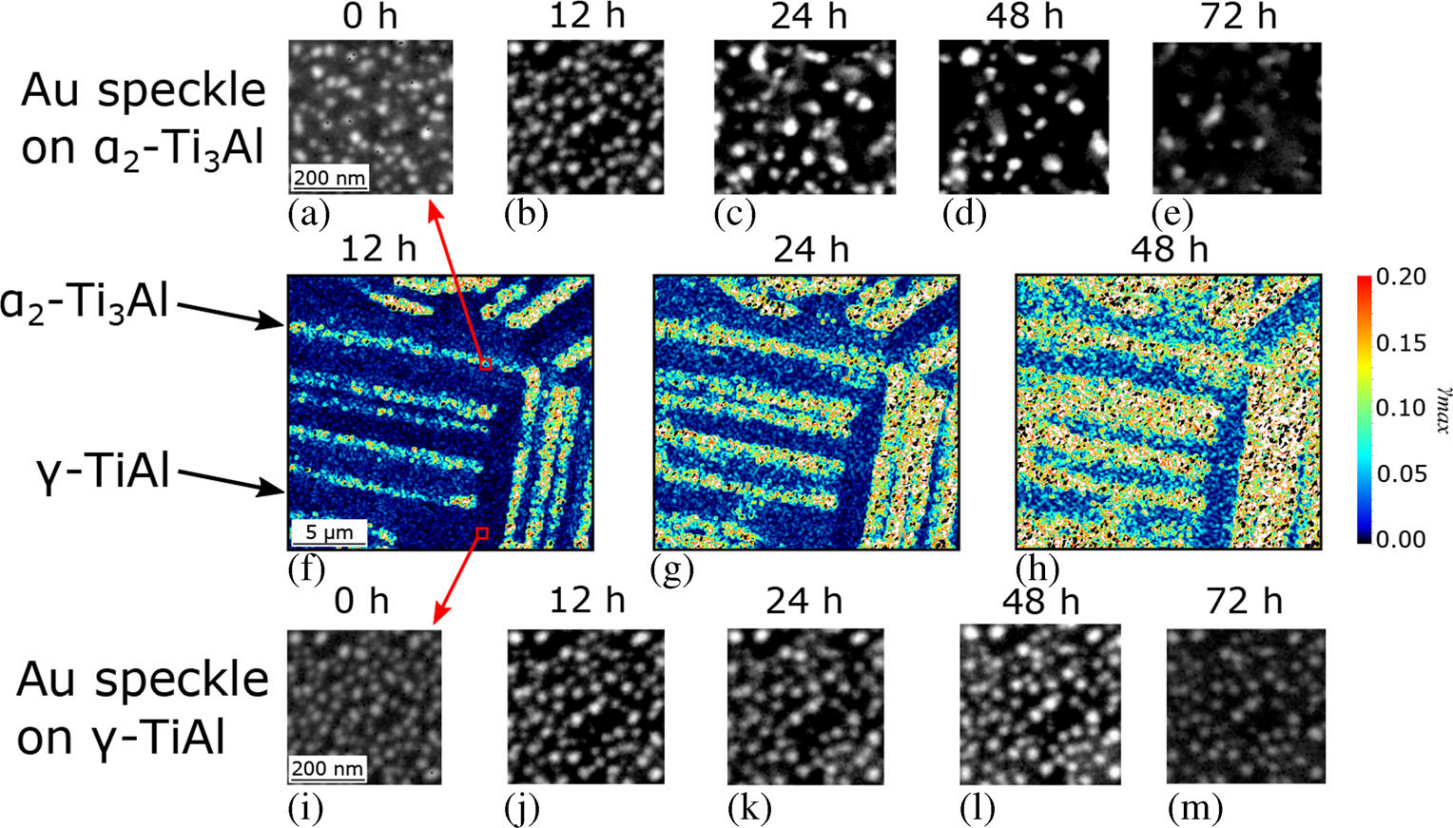
(**a**–**e**) and (**i**–**m**): backscatter electron images of the time progression of the high temperature Au speckle on α_2_-Ti_3_Al and γ-TiAl, respectively, upon holding in air at 700 °C. On the γ-TiAl phase significantly increased stability of the Au islands is observed compared to on α_2_-Ti_3_Al

Digital image correlation of repeatedly imaged regions of the high temperature speckle pattern gave a noise level in the maximum shear strain, Fig. 6(a–c), well below 1%, resulting from SEM scanning imperfections, for a subset size of 8 × 8 and hence a resolution of ~60 × 60 nm^2^. The island arrays generated by reconstruction may coarsen over time by coalescence due to island mobility [44]. To demonstrate the suitability of the pattern for use at high temperature, the same region was exposed for 1 h to successively higher temperatures, with electron imaging of the same region between heating steps, Fig. 7. Correlation of such images relative to the initial state revealed no deterioration of the backscatter imaged speckle pattern, Fig. 7(a–e) beyond the standard noise level. This occurred until the temperature reached 740 °C, despite considerable oxide growth between speckles, visibly obscuring the secondary electron images, Fig. 7(f–j). For monotonic loading tests, thermal stability for an hour was sufficient. However high cycle fatigue experiments required a pattern that was thermally stable for over 6 h. The long term stability of the pattern was therefore also investigated, Fig. 8. The pattern was found to coarsen exclusively on the α_2_-Ti_3_Al phase and the boride particles after 12 h, whereas Au speckles on γ-TiAl were stable for over 72 h. To further demonstrate the robustness of the reconstructed Au speckle pattern, a speckled sample was placed in an ultrasound bath of acetone for 20 min. Recorrelation of the same region, Fig. 6(d), showed no consequential change in the speckle pattern. Equivalent robustness of the room temperature-suitable speckle with regards to ultrasound cleaning was observed, Fig. 6(e).

Regions ~250 × 200 μm^2^ of the gauge sections of monotonic testing specimens underwent strain mapping. Equivalent tensile high cycle fatigue testing ensued. In order to achieve the desired spatial resolution, SEM imaging at 4000× magnification was necessary; backscatter imaging of the speckle with suitable low brightness, high contrast conditions, Fig. 4(a), produced sufficient compositional contrast of the gold to overcome contrast usually observed between α_2_-Ti_3_Al and γ-TiAl phases. With individual image widths of 32 μm, at 4096 × 3536 px^2^ resolution, 10 μs dwell and no integration, a 9 × 8 image array was required to cover the region of interest, with ~10% image overlap. Hence pattern imaging after each compression step could take several hours. In order to produce multiple strain maps for different testing conditions and stages of compression, automation of the imaging was necessary, and was performed using a custom script once low sample drift had been achieved and the sample carefully positioned to match the locations imaged before testing. Image correlation was performed in one user operation by loading the full list of images into DaVis, with sequential pairs undergoing correlation. Up to 30 passes per step were required, finishing with 25% subset overlap and normalised cross correlation for the final stage; image stitching ensued. A suitable minimum subset size for the patterns in the present study was determined by considering both the noise level of correlation of repeatedly imaged regions, Fig. 6(c), and the minimum spacing of shear bands within the material investigated. From this an 8 × 8 px^2^ subset was selected giving a resolution of ~60 × 60 nm^2^.

**Fig. 9:**
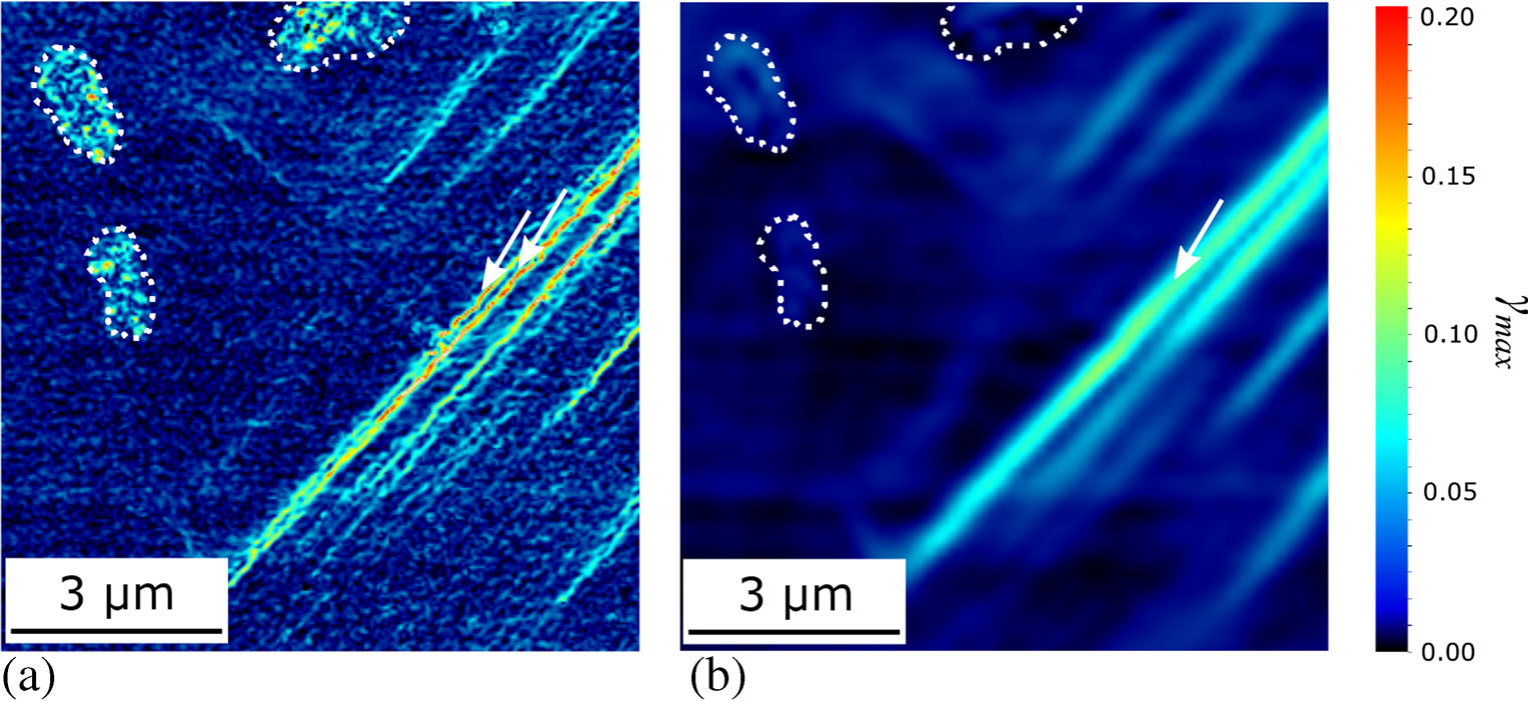
DIC strain mapping of the maximum shear stress after 3% compression at 700 °C of the Ti4522XD alloy with refined lamellae (air cooled condition). Correlation was undertaken with an (**a**) 8 × 8 px^2^ subset or a (**b**) 32 × 32 px^2^ subset; the micro-slip bands *arrowed* in (**a**) are less than 200 nm apart and are indistinguishable when a larger subset is used (**b**). Boride particles, indicated by *dotted outlines*, produce high noise with smaller subsets; this noise is removed when using a larger subset (**b**)

The TiAl alloy was tested in two microstructural conditions, both nearly fully lamellar, but with different mean lamellar thicknesses of 1.2 μm and 150 nm for the γ-TiAl layers. Strain mapping of the thinner lamellae condition using the speckle pattern and ~60 × 60 nm^2^ resolution presented above is shown in Fig. 9(a). The importance of such high resolution in identifying individual shear bands in a refined material is clear if compared to Fig. 9(b) for which the spatial resolution is a sub-micron ~0.25 × 0.25 μm^2^. Individual shear bands are now poorly distinguished. Other deformation features such as plasticity surrounding hard TiB_2_ particles were well resolved; this required both high accuracy and low noise of image correlation to resolve compound deformation components from the displacement fields such as the rotation of the underlying lattice [16]. However, for the coarser speckle pattern produced on boride particles, a 32 × 32 subset was most suitable, Fig. 9(b), to reduce noise from poor correlation of unsuitably small subsets.

**Fig. 10:**
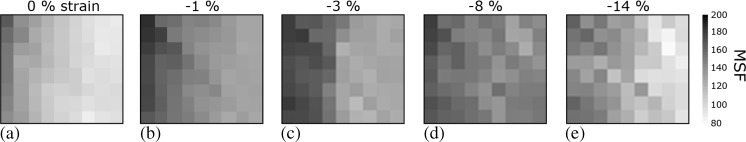
Greyscale mapping of the MSF value for each of the 72 consitutive images of the complete region of interest for DIC strain mapping, acquired at successive steps of compression of the Ti4522XD alloy at 700 °C

Compression of TiAl was performed in multiple steps, with the present example undergoing testing to strains of 1, 3, 8 and 14%. For the 72 images captured at each step, the MSF values were calculated, see Fig. 10. A first observation is that in all cases the MSF reduces from the top left, where imaging was initiated, to the bottom right, final image. No trend is observed between successive stages of compression.

Contrast and brightness degradation was not found to be an issue when testing at room temperature; that is to say, the Au speckle suffered no noticeable fine-scale damage as a result of electron imaging or general handling between testing steps. For the speckle tested at high temperature, the identical brightness and contrast conditions generally remained suitable between steps. Only after several cumulative hours (> 5 h) at temperature, owing to the requirement for cooling then reheating and stabilising between testing steps to allow for pattern imaging, was sufficient oxide found to have accumulated around the Au islands for slight manual adjustments in the brightness and contrast conditions of imaging to be necessary before initiation of automated imaging. Such small changes aimed to maintain similar speckle appearances to the initial state.

### Alternative Speckling Elements

**Fig. 11:**
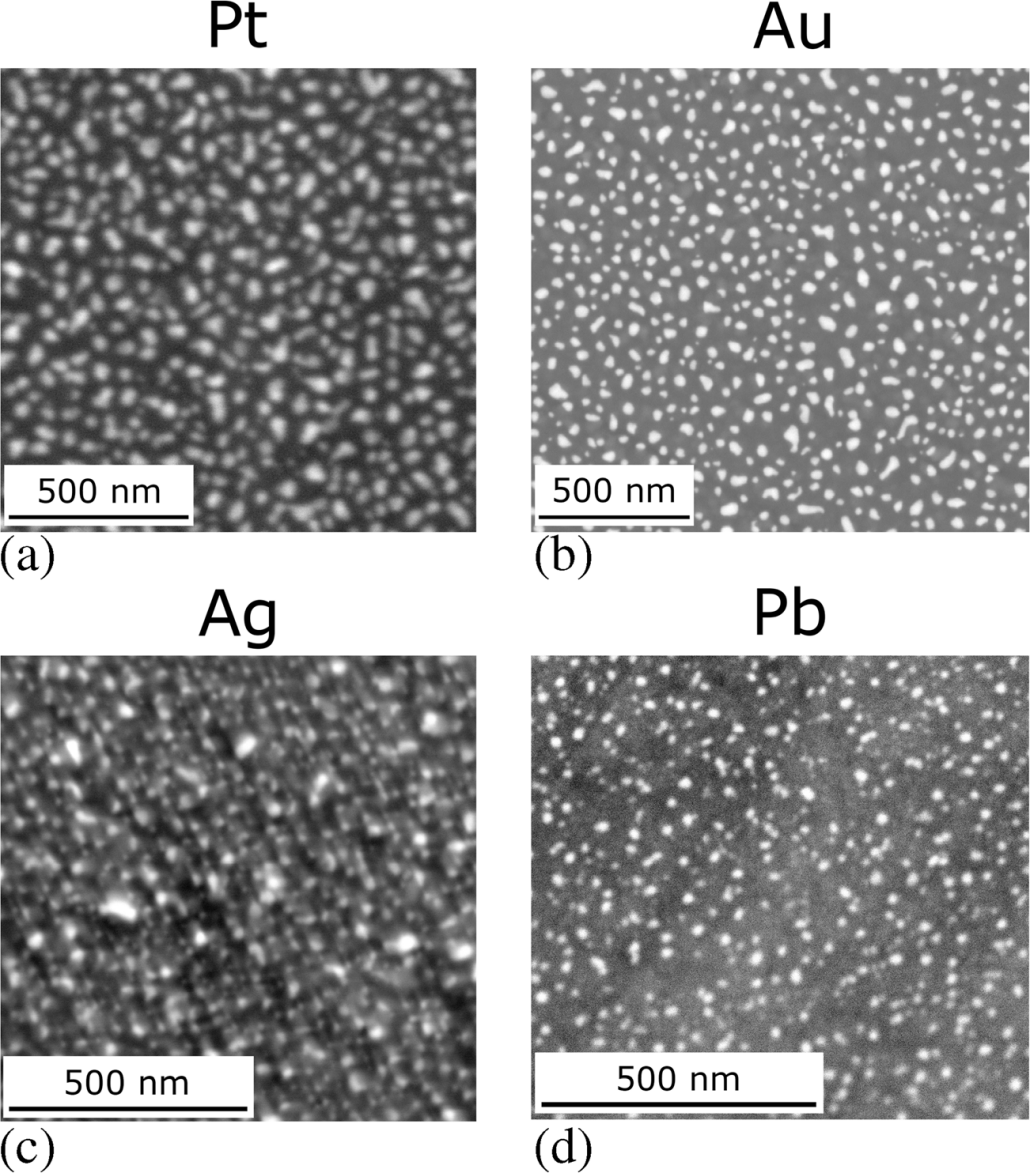
Backscatter electron images of reconstructed speckle patterns suitable nDIC strain mapping produced using four different elements: (**a**) Pt, for strain mapping at temperatures beyond 700 °C; (**b**) Au, as in Fig. 4(a); (**c**) Ag, obtained by heat treatment at 650 °C; and (**d**) Pb, obtained by heat treatment at 185 °C

To overcome the issue of Au island coarsening at 740 °C and above, Fig. 7, Pt was sputter coated onto the OPS vibratory polished TiAl surface. Reconstruction was achieved by the same approach as that for Au. An example of a remodelled Pt speckle pattern suitable for nDIC strain mapping is given in Fig. 11(a). The Pt speckle pattern was found to be stable up to 800 °C, at which point the oxidation of the current TiAl alloy became too excessive for surface strain mapping in air.

Alternatives to Au were also sought that might reconstruct at lower temperatures than 750 °C. Ag and Pb were both successfully remodelled to produce nano-scale island patterns in Fig. 11(c, d), after holding for short periods at 650 °C and 185 °C respectively.

## Discussion

### Advances and Benefits of the Gold Reconstruction Speckle Method

The sample surface patterning method presented here is demonstrated to be suitable for nanoscale DIC strain mapping, *nDIC*, up to temperatures in excess of 700 °C.

The use of heat treatments alone to reconstruct the Au thin film for DIC use has not been described before. Vapour-assisted remodelling has been used on various substrate materials such as glass [4], stainless steel [1, 45] and an Al-Si alloy [46], but was insufficient for reconstruction alone on TiAl for a sample temperature up to 300 °C. Within the context of thin film and device materials, extensive studies [47, 48] have investigated the effects of film thickness, temperature and time on island formation, as has the field of surface-enhanced Raman scattering [34]. The different reconstruction techniques also produce dissimilar Au particle morphologies; indeed, Luo et al. [34] reported globular, closely packed, Au particles following water vapour-assisted reconstruction, whereas in the present work the islands appear crystallographically facetted for those reconstructed in air, and globular but well-spaced for those reconstructed *in vacuo*. Such faceting reflects the exposure of specific crystallographic planes, and hence the islands achieving a more stable, low energy state, in a comparable manner to island growth during thin film deposition processes [49]. With respect to imaging of the speckle, the bimodal distribution of pixel intensities achieved is characteristic of successful speckle patterns identified elsewhere [38].

The robustness of speckle patterns is of significant practical importance, especially when mechanical tests are carried out *ex situ* and at high temperatures where contact lubricant may disperse across the sample surface. From the acetone ultrasound tests, the benefit of Au thin film reconstruction is clear, compared to other additive speckle methods such as SiO_2_ or Al_2_O_3_ colloid deposition where some speckle detachment upon such cleaning is immediate.

It is well reported that for colloidal SiO_2_ and metal nanoparticle deposited speckle patterns for SEM DIC strain mapping, the pattern is significantly non uniform across the specimen surface, displaying regions with much higher or lower densities of particles [15], with closely spaced, non-agglomerated spheres being desirable [2]. In contrast, no noticeable non-uniformities were observed for the speckle patterns presented in the current work across specimens multiple centimetres in size. This is undoubtedly a result of the uniformity of the initial Au sputter deposition preceding island reconstruction.

To achieve high resolution strain maps without phase bias in a multi-phase material, uniformity in speckle size and spacing is required in order for the same minimum subset size to be appropriate for all phases. Considering that Au film reconstruction conditions differ according to the substrate [4], achieving similar coarsening means finding the overlap in island growth parameter space on each phase, if it indeed exists. The detrimental effect of variable island coarsening on DIC is illustrated in Ti4522XD here: whilst an equivalent distribution, and hence strain mapping resolution, is achieved on both γ-TiAl and α_2_-Ti_3_Al, the minimum subset size required for TiB_2_ particles is to the detriment of the highest resolution necessary for shear band mapping in the refined lamellae condition, Fig. 9(b).

Though the aim of the current work was to generate a speckle pattern with suitable thermal stability for extended time periods for DIC strain mapping upon *ex situ* mechanical testing in fatigue, the pattern described here could also be used for *in situ* high temperature mechanical testing experiments. Furthermore, if use of a backscattered electron detector at high temperature is possible with the setup, then the compositional contrast of the pattern may exclude from the image surface oxides growing during the experiment. This is achievable by strongly negatively biasing the Faraday cage of an Everhart-Thornley detector to repel thermal electrons.

The spalling of the oxide layer intergrown between Au islands upon room temperature testing of the pattern generated in air, and hence loss of the speckle pattern, is consistent with observations [29] that TiAl forms an oxide that is ductile at high temperature, but brittle at room temperature. Au thin film reconstruction by *in vacuo* heat treatment is also new and gives a nearly oxide-free surface speckle. Preliminary high temperature tests at 700 °C with this pattern indicate it to be a promising alternative to the oxidised reconstructed Au speckle pattern, if mechanical testing is carried out *in vacuo*.

The quality of the patterns produced here, as evaluated using the MSF, indicates highly suitable speckle designs, with values consistently above maxima reported elsewhere [38]. The ~60 × 60 nm^2^ resolution (8 × 8 px^2^ subset) and corresponding noise level was found to be well adapted according to the SSDMSF and necessary to the identification of deformation features in the refined microstructure of the nearly lamellar TiAl alloy tested. The diminishing MSF as sequential imaging progresses is due to the loss of focus from slight inclination of the sample surface. Initial focussing in the centre of the region of interest before initiating automated imaging has effectively reduced the extent of such deterioration in the MSF value. The role of the experimentalist in obtaining a high MSF upon imaging is additionally seen in Fig. 10, where the brightness/contrast conditions selected to best overcome topographical effects in BSE imaging as deformation progresses cause the MSF values to vary non-uniformly between testing steps.

### Formation of Reconstructed Speckle Patterns Across the Temperature Range

The speckle patterning method developed in the current study, employing a maximum heat treatment temperature of 750 °C, has enabled the deformation mechanisms in TiAl between room temperature and 700 °C to be studied. In some cases, this heat treatment temperature for thin film reconstruction may be prohibitive to the use of Au; for example, in deformation studies on shape memory alloys, such a thermal cycle may significantly change the distribution of crystallographic variants initially present, whilst overaging of precipitates in 2000 and 7000 series aluminium alloy test-pieces may similarly occur [50]. It was apparent from the speckle development process that in the absence of vapour assistance, reconstruction should take place at a temperature above that of testing to ensure the stability of the pattern so that undue coarsening of the metal islands does not occur during testing. At a given temperature, the self-diffusion of pure metals varies inversely to the melting temperature [51]. Accounting for this, along with previous reconstructed thin film work [4] and the benefit of employing heavy speckling elements for high backscatter electron contrast, alternative speckling elements with lower melting points than Au, such as Ag or Pb, could be sought which will reconstruct at temperatures as low as 185 °C. This may be below the studied substrate material’s limiting temperature.

The progressively higher isothermal holds for 1 h in air of an Au speckled sample, Fig. 7, identified the limit of use of the Au speckle pattern produced in the present study to be below 740 °C. Strain mapping at temperatures above 700 °C was achieved with a reconstructed thin film of higher melting point Pt and was stable up to at least 800 °C.

In short, the use of alternative speckling elements enables the range of materials and test temperatures accessible to nDIC strain mapping to be extended relative to the limits of the Au speckle.

### Digital Image Correlation Equipment and Procedure

Beyond speckle application, the actual DIC method employed in the current study to generate high resolution, accurate strain maps combines recently established references by DIC pioneers such as SEM BSE imaging [52] and post-correlation stitching [43] in order to achieve a mapped area of ~250 × 200 μm^2^ covered by ~6500 × 5000 individual displacement vectors. The use of industry standard DIC software, along with a refined speckle pattern was vital if unambiguous measurements of local strain were to be obtained: as well as uniaxial strain along the image axes, *ε*_xx_ and *ε*_yy_, relatively noise-free maximum shear strain, max(*ε*_xy_), and in-plane rotation maps have been obtained. The latter are most relevant to identifying the underlying deformation micro-mechanisms such as slip bands and lattice rotation features, respectively [16].

## Conclusions

Gold remodelling is an effective method of producing a uniformly refined and dense random speckle pattern suitable for digital image correlation strain mapping at the nano-scale. Furthermore, it benefits from the significant advantage of using backscatter electron imaging in high contrast conditions, by which the imaging of oxides and other surface contamination is avoided, yielding a high quality image of the speckle pattern, with a high mean subset fluctuation. For such pattern quality quantification, the subset size dependent mean subset fluctuation, SSDMSF, introduced here may be a more practically useful indicator of pattern quality for a given target strain mapping resolution.

The standard vapour-assisted reconstruction approach to island speckle pattern formation from a metallic thin film was insufficient for remodelling on TiAl, as 300 °C was too low for more than a simple crazing of the film. Here the remodelling method has been extended by introducing heat treatments above the test temperature. This enabled both remodelling of the Au thin film to a suitably refined speckle pattern for nano-scale DIC strain mapping, and stability of the pattern at temperatures below 750 °C where both the cooperatively grown oxides and the reduced diffusivity of Au resist further reconstruction. Heat treatments at similar high temperatures with the Au thin film-coated sample encapsulated in quartz achieved comparably fine speckles without the surrounding oxide that would crack and spall along with the Au islands at room temperature.

The current heat treatments method therefore serves to increase the range of materials on which metallic thin film remodelling may be employed to produce a suitable speckle pattern for nDIC strain mapping, and to increase the temperature range of such strain mapping up to the operational temperatures of many structural high temperature materials. In the current work on a TiAl alloy, 250 × 200 μm^2^ surface regions were strain mapped at a resolution of 60 × 60 nm^2^ at 700 °C, with suitably low noise for compound strain and rotation values to be extracted and exploited.

## Electronic supplementary material

ESM 1 (PDF 63 kb)
