# Supplementary material for: Stable Speckle Patterns for Nano-scale Strain Mapping up to 700 °C
Source: Exp Mech. 2017 Jul 28;57(9):1469–82. doi: 10.1007/s11340-017-0317-8 (PMC6407731; doi:10.1007/s11340-017-0317-8)
Supplement: Supplementary file 1 — (PDF 63 kb) [file 11340_2017_317_MOESM1_ESM.pdf]

Stable speckle patterns for nano-scale strain mapping up to 700 °C

Experimental Mechanics

Thomas E. J. **Edwards**[a] Corresponding author (teje2@cam.ac.uk)

Fabio **Di Gioacchino**[a]

Helen P. **Springbett**[a]

Rachel A. **Oliver**[a]

William J. **Clegg**[a]

[a]*Department of Materials Science and Metallurgy, 27 Charles Babbage Rd, University of Cambridge, Cambridge CB3 0FS, UK*

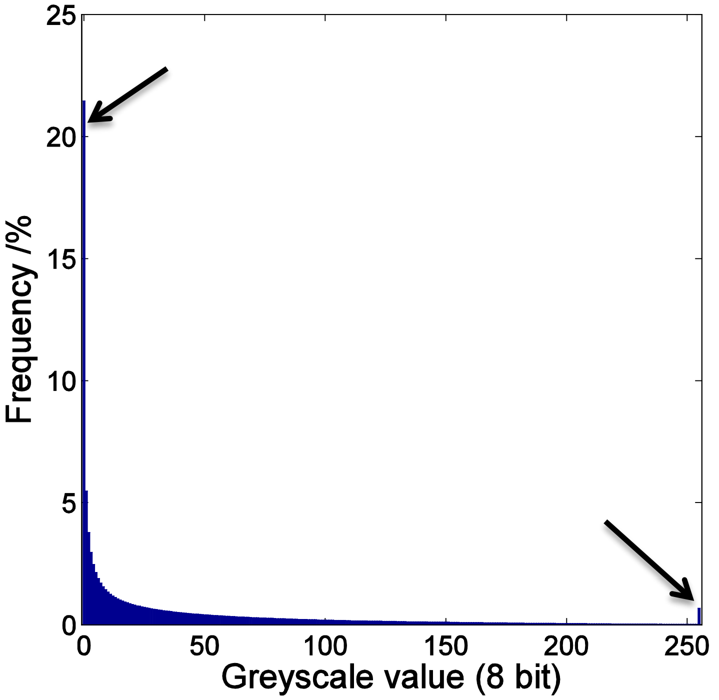

Histogram of pixel intensities for a backscatter image of the high temperature suitable reconstructed Au speckle pattern taken in standard conditions for DIC employed here. Arrowed are the two extremity maxima. Unlike commonly employed speckle patterns which possess a smooth, bimodal distribution of pixel intensities, the aim here was to push the peaks of the bimodes to extreme values to exclude imaging of the underlying dual-phase titanium aluminide and thermally grown oxides
